# Parity-Dependent Hemosiderin and Lipofuscin Accumulation in the Reproductively Aged Mouse Ovary

**DOI:** 10.1155/2018/1289103

**Published:** 2018-03-15

**Authors:** Ulises Urzua, Carlos Chacon, Renato Espinoza, Sebastián Martínez, Nicole Hernandez

**Affiliations:** Laboratorio de Genómica Aplicada, Departamento de Oncología Básica y Clínica, Facultad de Medicina, Universidad de Chile, Santiago, Chile

## Abstract

The progressive decline of the ovarian follicle pool leads to reproductive ageing. The latter is accompanied by age-related disorders, including various types of cancer. In fact, the highest rates of ovarian cancer (OC) occur at postmenopause while OC risk is significantly modulated by parity records during previous fertile life. We approached the age-parity relationship in the C57BL/6 mouse model and herein describe the presence of nonheme iron (hemosiderin) and deposits of the “age pigment” lipofuscin in reproductively aged mouse ovaries by applying conventional histochemical methods and autofluorescence. In addition, the 8-OHdG adduct was evaluated in ovarian genomic DNA. Both hemosiderin and lipofuscin were significantly higher in virgin compared to multiparous ovaries. The same pattern was observed for 8-OHdG. We conclude that nulliparity induces a long-term accumulation of iron and lipofuscin with concomitant oxidative damage to DNA in the mouse ovary. Since lipofuscin is a widely accepted senescence marker and given the recently postulated role of lipofuscin-associated iron as a source of reactive oxygen species (ROS) in senescent cells, these findings suggest a possible pathogenic mechanism by which nulliparity contributes to an increased OC risk in the postmenopausal ovary.

## 1. Introduction

Ageing is characterized by cumulative tissue and cell damage that impairs homeostasis and increases the risk of disease. Dysregulated oxidative stress concurrent with a depressed antioxidant defense is a predominant feature of such damage [1]. In the mammalian ovary, this age-related redox imbalance is paralleled by a steep decay in the quantity and quality of the follicular-oocyte reserve. This process culminates at menopause in women, a reproductive hallmark characterized by major systemic endocrine, metabolic, and inflammatory alterations, which together lead to higher risk of chronic pathologies including various types of cancer [2].

Regarding ovarian cancer (OC), both mortality and incidence increase significantly at early postmenopause. In addition, OC risk is modulated by reproductive history during former fertile life including use of oral contraceptives and parity records. Multiparity reduces while nulliparity increases OC risk [3]. The basis of this epidemiological evidence would be a tumor suppressor-like effect of progesterone and/or different degrees of ovulatory tear and repair stress to the ovarian surface epithelium (OSE), one of the candidate cell types where OC is thought to originate from [4].

Age-related changes of the postreproductive mammalian ovary comprise various biochemical and morphological alterations including a restricted steroidogenic capacity [5–7], decreased antioxidant gene expression coupled with increased oxidative damage [8], and residual expression of gonadotropin and steroid hormone receptors [9, 10] as well as increased stromal fibrosis, blood vessels remodeling, OSE invaginations, and epithelial inclusion cysts [11]. Relevant to the present study, nonheme iron and the “age pigment” lipofuscin have been also reported in aged mouse ovaries [12, 13]. However, to date, none of the above-described age-related changes have been linked to parity history. In the present work, we extend a previous study on female C57BL/6 mice at early postmenopausal age equivalent (20 months old), which were maintained in nulliparous and multiparous regimens [14]. Results shown below in detail suggest that nulliparity, the condition associated to increased OC risk, promotes the accumulation of ferric iron (hemosiderin) and lipofuscin deposits, with concomitant oxidative DNA damage. We discuss the possible mechanisms by which this parity-dependent phenomenon might initiate during fertile life and how it would be implicated in OC pathogenesis at postreproductive age.

## 2. Materials and Methods

### 2.1. Animals and Sample Collection

Female C57BL/6 mice were handled under protocol number 0536 previously approved by the Bioethics Committee, Faculty of Medicine, University of Chile. Care and monitoring of the two experimental groups, virgin and multiparous, has been described in detail recently [14]. At the ages indicated below, one subset of animals was euthanized to collect ovaries for histochemical and autofluorescent studies, and a second subset was euthanized for extraction of ovarian DNA and subsequent 8-hydroxy-d-guanosine (8-OHdG) assays. Multiparous animals had at least 2 litters (range 2–7). Mean number of litters of the multiparous group was 3.8.

### 2.2. Histochemical Methods

Dissected ovaries (*n* = 10 virgin; *n* = 10 multiparous; mean age 20.5 ± 1.7 months old) were fixed in 1% p-formaldehyde, pH 7.2 for 8 hrs at room temperature, and embedded in paraffin. Whenever possible, the oviduct and the distal portion of uterine horn were included. Depending of the tissue sample size, 3 to 6 sections, 5 *μ*m thickness each, were placed in a single slide. Prior to the histochemical staining, sections were deparaffinized with xylol and rehydrated with ethanol solutions of decreasing concentrations (95–70–50%) followed by distilled deionized water. Hematoxylin-eosin (HE) stain was done according to routine protocols. Perls stain to determine hemosiderin (nonheme ferric iron) was done by incubating sections in a freshly prepared solution of 2.5% potassium ferrocyanide and 2.5% HCl for 30 min. After a brief wash with distilled water, 0.1% safranin counterstain solution was applied for 10 seconds. To demonstrate type I collagen fibers, the van Gieson method was conducted by first staining nuclei for 5 min with a freshly prepared 0.5% Weigert's ferric hematoxylin solution, then a water rinse for 5 min followed by incubation with a solution of 0.1% Ponceau S and 0.02% acetic acid in saturated picric acid for 5 min. The sections stained with HE, Perls, and van Gieson were dehydrated in ethanol solutions of increasing concentration followed by xylol incubation and were mounted in Entellan® (Merck, Germany). The lipophilic Sudan Black B (SBB) stain was used to demonstrate the presence of lipofuscin. This was carried out in slides dehydrated up to 70% ethanol. A fresh saturated ~1% SBB solution was prepared in 80% ethanol, stirred for 2 hr, and filtered through paper. Sections were incubated with this solution for 5 min and sequentially washed in 70% and 50% ethanol and distilled water. Safranin counterstain was applied for 5 min. Slides were mounted in glycerin and immediately imaged. All staining protocols were performed at room temperature.

### 2.3. Autofluorescence

Tissue sections were deparaffinized, rehydrated, and air-dried to be analyzed in a ScanArray Lite fluorescent scanner (Perkin Elmer, USA) using 543 nm as excitation wavelength, with resolution of 5 *μ*m and 35–50% of laser power. TIFF images were captured and pseudocolored (rainbow palette) by using the ScanArray Express software (Perkin Elmer, USA).

### 2.4. DNA Isolation and 8-OHdG Immunodot-Blot Assays

Once dissected, ovary samples were immediately homogenized with QIAshredder microcentrifuge spin columns (QIAGEN, USA). Homogenates were extracted with the AllPrep kit (QIAGEN, USA) following instructions from the manufacturer. RNA and protein fractions were saved and frozen for further analysis. DNAs were quantified at 260 nm in an Epoch spectrophotometer (Biotex, USA). The immunodot-blot assay was based on Shi et al. [15] with modifications as follows: aliquots of 100 ng of genomic DNA were denatured at 95° for 5 min, chilled on ice, and deposited onto Amersham Hybond-N+ nylon membranes (GE Healthcare, UK). DNAs were covalently attached for 30 secs in a UVC Crosslinker (Hoefer Scientific, USA). Membranes were then incubated in blocking reagent (Cod NIP552 GE Healthcare, UK; prepared in 4% PBS) for 1 hr at room temperature with shaking. The monoclonal 8-OHdG (15A3) antibody (Santa Cruz Biotechnology, USA) was diluted 1 : 1000 in PBS and applied to membranes for 16–18 hrs at 4°C. After 5 washes with PBS, 3 min each, the peroxidase-coupled anti-mouse IgG/anti-rabbit IgG biotinylated secondary antibody from the RTU Vectastain Universal, ABC kit (Vector Laboratories, USA), was diluted 1 : 2 with PBS and added to membranes for 45 min at room temperature. Further, 5 washes in PBS, 3 min each, were done, and the Streptavidin Reagent of the above-mentioned ABC kit was applied for 30 min. Finally, 200 *μ*L of the 1 : 2 buffer-diluted SuperSignal West Femto Chemiluminescent Substrate (Thermo Scientific, USA) was used to detect the dot-blot signals by incubating 5 min at room temperature protected from light. Membranes were scanned in a C-DiGit® blot scanner operated through the Image Studio Digits v3.1 software (LI-COR, USA). Magnitudes of chemiluminescent signals were directly quantified with this software. An ovarian genomic DNA from a young (3 months old) mouse oxidized by means of an *in vitro* Fenton reaction mixture was used as the positive control. PBS was used as the negative control.

### 2.5. Image Analysis and Statistics

Autofluorescent and histochemical TIFF images were analyzed with the ImageJ1 software [16]. Briefly, using the HE stain as a guide, the whole ovarian area delineated by the OSE was marked with the *Freehand* key. The image was then converted to a grayscale (RGB stack), and based on its histogram, a gray threshold was set for each individual image in consistency with the original positive stain. Autofluorescence, Perls, and SBB quantification were done in the red channel. Autofluorescent images were previously reversed with the *Invert* function. The areas of positive signal were obtained with the *Measure* function and were expressed as a percentage of the total ovarian area initially defined. Statistically significant differences between conditions across various staining methods, autofluorescence, and dot-blot assays were addressed with the GraphPad Prism 5.0 software using the nonparametric Mann–Whitney test with *p* < 0.05. Results are plotted as mean ± standard error of the mean.

## 3. Results and Discussion

### 3.1. Hemosiderin in Aged C57BL/6 Ovaries

**Figure 1:**
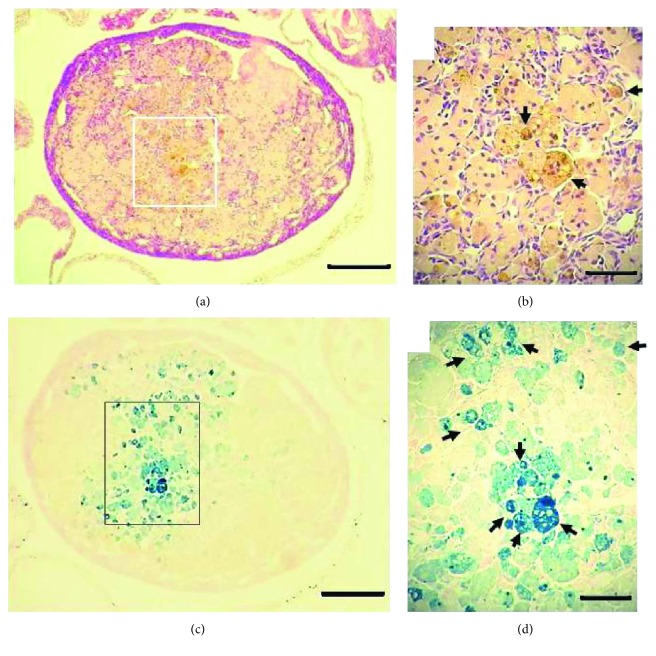
Hemosiderin in the aged C56BL/6 ovary. HE stain (a, b) and Perls stain (c, d) of a virgin ovary 20.6 months old. Arrows in (b) indicate brown-yellowish granules of hemosiderin. Arrows in (d) show hemosiderin laden macrophages (HLMs). Bars in (a) and (c) = 200 *μ*m; bars in (b) and (d) = 20 *μ*m.

Trace iron amounts are necessary for p450 cytochrome and FeS center containing enzymatic activities of the ovaries, mostly follicular steroid synthesis. Altered iron metabolism has been linked to polycystic ovarian syndrome [17], endometriotic cysts in human ovaries [18], and other less common ovarian pathologies. The aged mouse ovary has been reported to accumulate nonheme iron [12], but no link to parity history is suggested in the literature to date. Figures 1(a) and 1(b) show the HE stain of a virgin ovary, 20.6 months old, in which enlarged globular cells display brown-yellowish cytoplasmic granular stain matching the typical description of intracellular hemosiderin deposits. In agreement with the observations in the ovaries of 12 months (52 weeks) old C57BL/6 [12], 22 months old CD1, and 14–17 months old CB6F1 mice [19], these enlarged cells could be foamy multinucleated “giant” macrophages surrounded by flattened fibroblasts. The classical Perls' Prussian blue (hereafter referred to simply as Perls) method shown in Figures 1(c) and 1(d) provided an enhanced detection of ferric iron by extending sensitivity to a larger area of the ovary, although not every enlarged macrophage cluster in Figure 1(b) is shown loaded with hemosiderin in Figure 1(d). This pattern suggests that iron uptake and accumulation may be selective to certain subpopulation of ovarian macrophages. The markedly Perls stained cells shown in Figure 1(d) might correspond to hemosiderin laden macrophages (HLM) that in other tissues are primarily formed as a consequence of red blood cell (RBC) phagocytosis [20]. Though events requiring RBC clearance have not been systematically studied in the mouse ovary, the postovulatory microhemorrhages preceding *corpus luteum* (CL) formation from follicle remains—sometimes called *corp*u*s hemorrhagicum*—could be a source of iron from RBCs that after lifetime ovulations would accumulate with age in the form of HLM. Another possible hemosiderin source would be the leakage of RBC from permeable blood vessels in the aged ovary. Indeed, vascular changes have been described in human postmenopausal ovaries [11]. In the case of women, endometriosis can be a source of RBC iron in the ovary and has been linked to increased risk of developing clear-cell and endometrioid epithelial OC [21].

**Figure 2:**
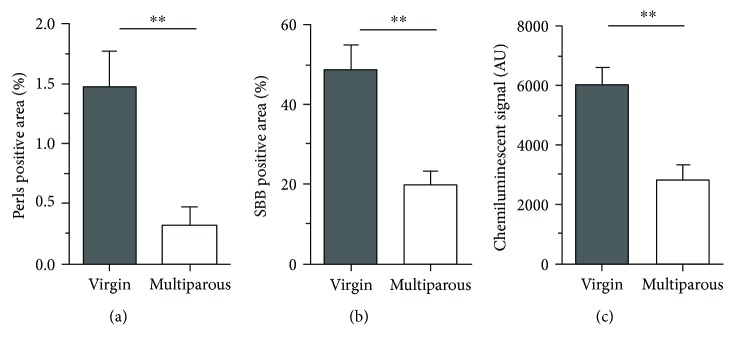
Semiquantification of hemosiderin, lipofuscin, and 8-OHdG in aged ovaries. Percentages of positive signal areas of (a) Perls, *n* = 8 per group, and (b) SBB, *n* = 7 per group, relative to the total ovarian area as extracted with the ImageJ1 software. (c) 8-OHdG in isolated genomic DNA, *n* = 6 per group; AU = chemiluminescent arbitrary units measured with the Image Studio Digits software (for details, see Materials and Methods). ^∗∗^*p* < 0.005, nonparametric Mann–Whitney test.

A variant pattern of ovarian hemosiderin deposits observed in the present work was less diffuse, that is, confined to macrophage clusters of stronger Perls signal, commonly seen in the ovarian cortex (not shown). More importantly, the signal intensity, extension, and number of hemosiderin deposits were higher in aged virgin (nulliparous) compared to aged multiparous ovaries. To accurately quantify this observation, the Perls-positive signal area as a percentage of total ovarian area was measured in the ovaries of the two conditions (see Materials and Methods).  Figure 2(a) shows that nonheme iron (hemosiderin) amount was significantly higher in nulliparous compared to multiparous ovaries. Nulliparity means no CL rescue over reproductive life. In fact, during a nonfertile cycle, the C-C motif ligand 2 (CCL2) cytokine stimulates macrophage infiltration promoting CL regression with loss of progesterone and luteotrophic prostaglandin-E synthesis by luteal cells [22]. Therefore, assuming a postovulatory hemorrhagic RBC origin of ovarian iron during ageing, the multiparous ovary was subjected to ovulatory pause periods during which insoluble iron in hemosiderin might have been released and used to synthesize p450 cytochrome enzymes supporting progesterone synthesis by the CL during pregnancy. In this regard, it would be interesting to determine if the overall macrophage infiltration differs between studied conditions. However, as described in the primate ovary, other immune cells including neutrophils, natural killer, and T- and B-lymphocytes participate in luteolysis [23] and eventually could interact with and modulate the fate of HLM.

### 3.2. Lipofuscin in Aged C57BL/6 Ovaries

**Figure 3:**
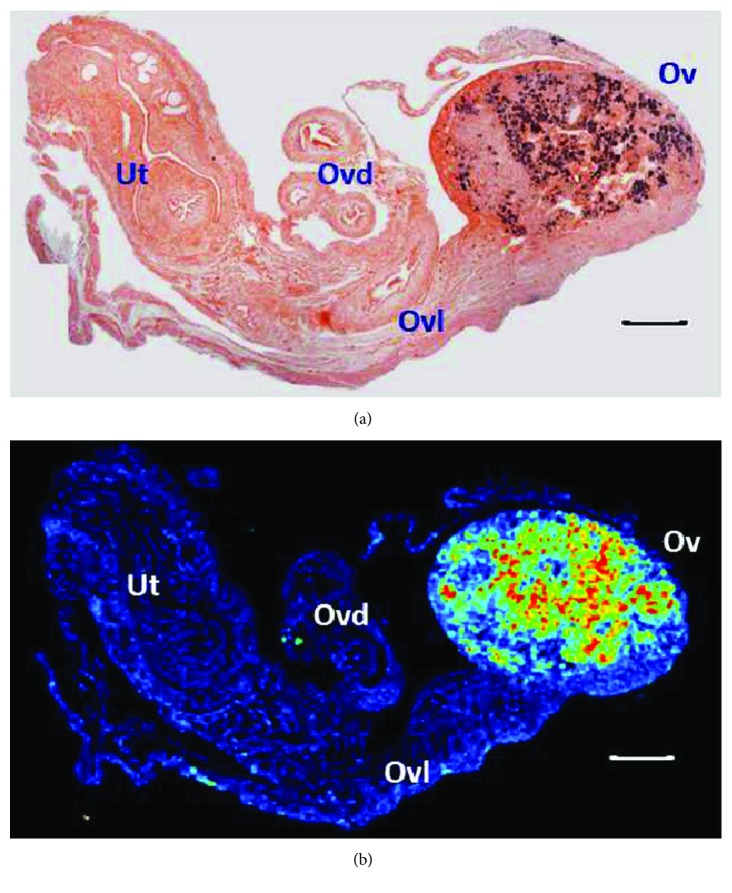
Lipofuscin in the aged C56BL/6 ovary. SBB stain (a) and 543 nm green laser autofluorescence (b) of a 20.9 months old virgin ovary. Ut = uterine horn; Ovd = oviduct; Ovl = ovarian ligament; Ov = ovary. Bars in (a) and (b) = 200 *μ*m. Image in (b) was obtained with the rainbow palette.

Also referred to as ceroid or “age pigment,” lipofuscin is an intracellular, cross-linked, oxidized heterogeneous (protein-lipid-carbohydrate and metal traces) substance formed as a result of impaired lysosomal and/or proteasomal activity [24]. Lipofuscin increases with age and is considered a marker of senescent cells in various tissues [25]. As shown in Figure 3, we used both autofluorescence and SBB staining to demonstrate lipofuscin in mouse ovaries >20 months old. SBB is well known to specifically identify lipofuscin thereby allowing detection of senescent cells [26, 27]. We observed a good correspondence between the two methods, that is, autofluorescence colocalized in a significant extent with the SBB stain. Notably, no signal was detected in the oviduct, ovarian ligament, or uterine horns, indicating that lipofuscin accumulates exclusively in the aged mouse ovary (Figure 3). This finding supports the notion that the ovary undergoes earlier biological ageing relative to the other major organs [28]. Upon image quantification, we found that virgin ovaries showed a significantly higher level of lipofuscin relative to multiparous ovaries (Figure 2(b)). Similar to hemosiderin, a relationship between lipofuscin and parity has not been reported to date. It sounds reasonable to think that virgin ovaries experience an uninterrupted and repetitive phagocytic demand over apoptotic cells derived from atretic follicles and from nonfertile, regressing CL. As lysosomal efficiency is reduced with age, the result is an overload of incompletely digested cellular debris that polymerizes and oxidizes. In contrast, the ovulatory pause during pregnancies in multiparous mice reduces the atretic and luteal phagocytic load. In addition, as oxidative damage is lower in the multiparous ovary (see below), lysosomal activity would be preserved and lipofuscin formation minimized. Importantly, in a B6C3F1 strain OC mouse model with chemically induced estropause, the analysis with two-photon excited fluorescence microscopy and histochemical methods revealed significant age and disease-dependent lipofuscin content in the ovary [13]. Furthermore, lipofuscin—referred to as ceroid—colocalized with diaminobenzidine- (DAB-) enhanced Perls stain in ovoid macrophages of middle age (8 months old) and enlarged macrophages of aged (12 months old) mouse ovaries [12], confirming an additional role of macrophages in both iron and lipofuscin age-dependent storage further than their participation in folliculogenesis, ovulation, corpus luteum dynamics, and vascular integrity [29, 30]. Given the limited sensitivity of the conventional Perls method used in the present study when compared to the perfused, DAB-enhanced procedure by Asano [12], we were unable to determine a precise hemosiderin-lipofuscin colocalization in our samples. In this regard, the complex distribution pattern of the diverse ovarian macrophage subsets [31] in addition to their relatively unmodified age-dependent fraction relative to age [19] suggests that their hemosiderin and lipofuscin storage capacities might be different.

### 3.3. Genomic DNA Damage in Aged Ovaries

As mentioned above, ageing is characterized by disruption of the redox balance due to impaired antioxidant defense, increased ROS production, or both simultaneously. We hypothesized that stores of iron and lipofuscin in the aged mouse ovary contribute to oxidative damage of genomic DNA.  Figure 2(c) shows that 8-OHdG levels in DNA of virgin ovaries were higher than in multiparous ovaries, resembling the patterns of hemosiderin and lipofuscin. Hemosiderin is a low-soluble ferritin aggregate containing mostly ferric iron but also significant amounts of ferrous iron [32]. Through the Fenton reaction, ferrous iron can generate highly reactive hydroxyl radicals inflicting damage to cell components. In the mammalian ovary, oxidative damage to lipids, proteins, and nucleic acids has been mostly attributed to a depressed antioxidant defense concomitant to the progressive decline of the ovarian reserve, the underlying cause of menopause [8, 33]. The primary determinant of this disrupted redox balance during normal ovarian ageing has not been elucidated. Our findings suggest that iron and lipofuscin could contribute to higher oxidative stress in the aged virgin compared to the aged multiparous mouse ovary. In this regard, it would be interesting to see if the various toxicant agents as well as the genetically modified mouse models targeting the follicle reserve [34] also induce iron and lipofuscin accumulation, further increasing oxidative stress. Importantly, as ovarian mitochondrial function decays with age affecting oocyte and follicle homeostasis [35], iron associated lipofuscin has been proposed as a mitochondrial-independent ROS source. Höhn et al. showed that synthetic lipofuscin was able to uptake ferrous iron, and this complex showed proteasome inhibition and ROS-forming capacities [36]. Furthermore, as these authors determined that iron uptake by lipofuscin was saturable at ~1.3 *μ*moles Fe(II)/mg of synthetic lipofuscin, a minimal amount of stoichiometric ferrous ion *in vivo* would be required to generate a ROS-producing redox-active surface. This idea is consistent with the divergent magnitudes of hemosiderin and lipofuscin areas in aged mice ovaries of our study (Figures 2(a) and 2(b)).

## 4. Conclusion

The risk of OC increases during the postmenopause and decreases with parity and oral contraceptive use during prior fertile age. Systemic exposure to progesterone (progestins) and decreased ovulatory cycles characterize these reproductive conducts. Here we show evidence of differential accumulation of both hemosiderin and lipofuscin in the postreproductive mouse ovary (>20 months old) according to its previous parity history. Aged virgin—nulliparous—ovaries contained significantly higher amounts of the two pigments, a finding that correlated with increased levels of oxidative DNA damage. Based on morphology and studies by other authors, these two residual substances would be contained in multinucleated enlarged macrophage clusters. Provided that ferric iron present in hemosiderin is reduced to ferrous iron, the diffusion of the latter across ovarian stroma and its uptake by lipofuscin would be an important source of endogenous ROS. The precise mechanism by which hemosiderin and lipofuscin accumulates in the ovary as a function of parity and age deserves further research.
